# Structured hybrid photodetectors using confined conducting polymer nanochannels†

**DOI:** 10.1039/d3na00485f

**Published:** 2023-10-18

**Authors:** Sukanya Das, K. H. Girish, N. Ganesh, K. S. Narayan

**Affiliations:** a Chemistry and Physics of Materials Unit, School of Advanced Materials, Jawaharlal Nehru Centre for Advanced Scientific Research Bengaluru – 560064 India narayan@jncasr.ac.in

## Abstract

We design and fabricate hybrid organic inorganic perovskite photodetectors that utilize hole transport layer poly(3,4-ethylene dioxythiophene):poly (styrenesulfonate) PEDOT:PSS confined in alumina nanocylinders. This structural asymmetry in the device where the alumina nanopore template is partially filled with PEDOT:PSS provides features that improve certain device characteristics. The leakage component of the current in such devices is considerably suppressed, resulting in enhanced responsivity and detectivity. The funneling aspect of the photogenerated charge carrier transit ultimately leads to fast detectors as compared to conventional perovskite detectors.

## Abstract 2

We design and fabricate hybrid organic inorganic perovskite photodetectors that utilize hole transport layer poly(3,4-ethylene dioxythiophene):poly (styrenesulfonate) PEDOT:PSS confined in alumina nanocylinders.

## Graphical abstract

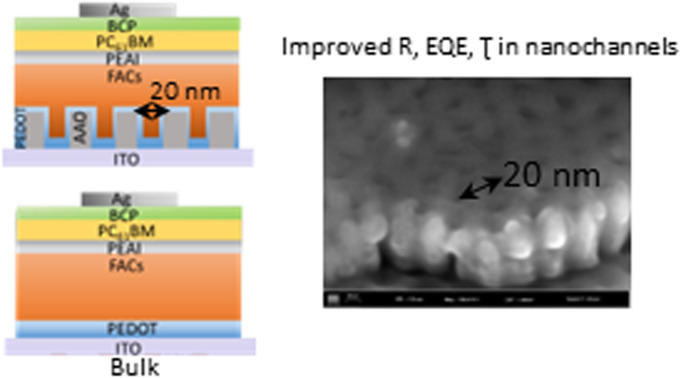

## 1 Introduction

Hybrid perovskite-based photodetectors hold tremendous promise for broadband optoelectronic applications due to the large optical absorption coefficient over the ultraviolet to infrared spectral range and long diffusion lengths. There has been a significant effort to understand the source of hysteresis in current–voltage response, grain boundaries, type of charge defects and ionic migration. The improved understanding of the physics and chemistry of the transport mechanisms in perovskites has led to rapid enhancement of the device performances with striking progress.^1–11^ Traditional photodetectors that convert optical signals to electrical signals are primarily based on InGaAs, GaN, and Si that require elaborate film growth techniques.^12–21^ More recently solution processible semiconductor-based hybrid perovskites and organic polymers have shown promise in applications like optical communications, imaging, chemical and biological sensing. However, direct gap solution processible semiconductors with reduced complexity in manufacturing, flexible form factors accompanied by high absorption and carrier lifetime are always desirable over disordered materials in these applications.^22–25^

A photodetector device architecture that incorporates a patterned alumina layer provides robust features that considerably reduce parasitic currents while enhancing the performance parameters. A key ingredient in the device is the nanoconfined PEDOT:PSS with significant electrical conductance along the nanochannels aligning with the transport of the photogenerated carriers. The cylinder walls of nanochannels provide an inherent boundary to the otherwise exposed thin-film junction of the PEDOT-perovskite layer. This integrity of the device structure ensures minimum pinhole formation. Since the HTL in the inverted devices is the first polymer layer on which the light is initially incident, it plays an important role in the device performance and requires good light transmission, energy level alignment and charge transport properties.^26,27^ The growth of perovskite films, grain sizes and defect density depend also on the interfacial surface properties of the HTL. Nanoconfined perovskites have been utilized in earlier studies^25,28,29^ which have shown promising device properties and improved stability. There are many stable HTL materials,^30–32^*viz.*, PTAA, NiOx, CuI and CuSCN which have been studied to date, but PEDOT:PSS remains as one of the best candidates owing to its excellent light transmittance, wettability for perovskite precursors and conductivity of charge carriers.^33–35^ Parallelly, the low work function ∼5.2 eV, insulating counterpart PSS and high acidity are a few drawbacks of PEDOT:PSS. The hygroscopic nature of PEDOT:PSS also accelerates the perovskite degradation. There have been many studies on the efforts to modify PEDOT:PSS as the HTL in terms of reduction of the hole injection barrier, conductivity enhancement or interfacial surface roughness.^36,37^ PEDOT:PSS extracts the photogenerated exciton-separated holes through the HTL and perovskite interface and transfers them to the respective electrode. Thus, the modification of the HTL is one of the crucial factors for the enhanced performance of the photodetectors. The structural asymmetry of the PEDOT:PSS/perovskite device also mitigates the ion accumulation at the interface of the HTL/active layer to ensure enhanced device performances.

With the advent of mixed cation and mixed anion perovskites, researchers have achieved better stability, reproducibility and higher yields.^28,38^ It has been observed that formamidinium lead triiodide, FAPbI_3_, shows better stability than methylammonium lead triiodide, MAPbI_3_ perovskite, but this comes along with an undesired photo-inactive yellow δ-FAPbI_3_ phase. The yellow phase is then prevented by combining MAPbBr_3_ and FAPbI_3_. Further, the addition of small cesium cations assists α-FAPbI_3_ black phase crystallization and has opened a class of materials FA_1−*x*_Cs_*x*_Pb(I_1−*y*_Br_*y*_)_3_ that shows improved thermal and moisture stability with no undesired phase transition within the operating ranges of temperature.^39–47^ The ease of controlling the stoichiometry and the I/Br and FA/Cs ratios in mixed perovskites help to tune the bandgap of the materials. The required cesium content is dependent on the device processing conditions.^48,49^ An optimized formula FA_0.95_Cs_0.05_Pb(I_0.83_Br_0.17_)_3_ (FACs) has been adopted in the present work in fabricating a vertical HOIP photodetector.

The morphology, conductivity, crystallinity and degree of order in PEDOT-derivatives as a function of structural confinement have been found to be strongly dependent on transport dynamics in our previous studies.^50–52^ Our understanding of the enhancement in surface and electronic properties of PEDOT derivatives has been employed in fabricating a HOIP photodetector device in this work. The commercial PEDOT:PSS usually comes in different grades with different PSS ratios which effectively shows different work functions, viscosity, conductivity and so on. In this work, we have used a mix of two grades AI4083 and PH1000 which have shown higher magnitudes of photocurrent as compared to an untreated PEDOT:PSS as a HTL. A 50–60 nm thick PEDOT layer is introduced in alumina scaffolds of thickness of around 200 nm with a channel diameter of 20 nm. The remaining length of nanochannels is occupied by the perovskite layer along with a bulk layer (∼300 nm) above the nanochannels. The devices without alumina are analysed as control devices. In this paper, we have studied the enhanced photodetection properties due to the introduction of structural confinement of the HTL along with a portion of the perovskite layer. We have compared the nanochannel device with that of the control device without the alumina in terms of responsivity, EQE, detectivity and response speed.

## 2 Materials

Device fabrication of the HOIP involved the use of Indium tin oxide (ITO) as the bottom electrode with a 7 Ω □^−1^ specific sheet resistance and shaped in a particular mask as shown in ESI S1.† PEDOT:PSS is used as a hole transport layer where two grades of polymer have been used, *viz.*, AI4083 and PH1000 with PEDOT/PSS ratios as 1 : 6 and 1 : 2.5, respectively. Formamidinium iodide >99.99% (FAI) from Greatcell Solar, cesium iodide 99.999% (metals basis) (CsI) from Alfa Aesar, 99.99%, trace metals basis lead iodide (PbI_2_) from Tokyo Chemical Industry and 99.999% (metals basis) lead bromide (PbBr_2_) from Thermo Fisher Scientific have been used for the preparation of perovskite solution. 99.8% anhydrous *N*,*N*-dimethylformamide (DMF), ≥99.9% dimethyl sulfoxide (DMSO) and anhydrous anisole, 99.7% were purchased from Sigma-Aldrich. Phenethylammonium iodide (PEAI) from Greatcell Solar, [6,6]-phenyl-C61-butyric acid methyl ester (PC_61_BM) and bathocuproine (BCP) from Luminescence Technology Corp. were used.

### 2.1. Sample preparation

HOIP nanochannel films: 2.5*2.5 cm^2^ ITO substrates etched in a specific pattern have been used as transparent bottom electrodes for the inverted structure. Standard cleaning processes are adapted by subsequent ultrasonic washing with acetone, isopropyl alcohol, and deionized water. Cleaned substrates are made hydrophilic using RCA, *i.e.*, ammonia:hydrogen peroxide:water (1 : 1 : 5 mixture) and air plasma treatment. PMMA-supported AAO membranes are adhered to the ITO substrates with a stepwise acetone bath. The ITO/AAO samples are once again plasma treated. Two grades of PEDOT:PSS, AI4083 and PH1000 are mixed in a 1 : 1 ratio and stirred for 2–3 h. This solution will be named PEDOT:PSS mix' in the remaining part of the manuscript. The solution is filtered using 0.45 μm PVDF disposable filter paper and spin coated on cleaned ITO/AAO substrates at 4000 rpm for 60 s and 10 s of ramping. The films are then heated at 140 °C for long hours to remove aqueous solvent from the nanochannels. The films are cooled to room temperature before spin coating the perovskite layer inside a nitrogen glovebox. 1 M solution of FA_0.83_Cs_0.17_Pb(I_0.9_Br_0.1_)_3_ is prepared by mixing 783.7 mg of PbI_2_, 285.46 mg of FAI, 110.1 mg of PbBr_2_ and 88.34 mg of CsI in 2 ml of 4 : 1 ratio of DMF : DMSO solvent. The solution is stirred for 15 min at 500–600 rpm at 70 °C followed by further stirring overnight without heating. This solution is drop-cast on PEDOT:PSS/AAO substrates and given a waiting time of 40 s before spin coating at 1000 rpm for 10 s followed by 6000 rpm for 35 s. In the last 15 s of spinning, antisolvent anisole is added to the substrates. After spin coating, the films are then immediately transferred to a preheated surface of a heater at 100 °C for 1 h. Slow flushing of the glovebox for 15 min is performed after the annealing is completed to remove any residual vapour of DMF and DMSO. The passivation layer PEAI is then spin coated at 5000 rpm for 30 s and 2500 rpm ramping and kept under ambient conditions in a glovebox for 1–2 min. PC_61_BM, which is the electron transport layer, is spin coated at 2000 rpm for 30 s and stored inside a glovebox in a covered Petri dish for overnight solvent annealing. Next, the films are heated at 100 °C for 30 min. BCP solution is coated at 4000 rpm for 30 s and heated at 100 °C for 1–2 min. The substrates are allowed to cool to room temp and transferred to a metal mask for Ag electrode deposition. Effectively, 120 nm of Ag is coated following a deposition rate of 0.1 Å s^−1^ inside the physical vapour deposition chamber. A similar process was followed for control devices without the AAO membranes.

## 3 Methods

A Keithley 2400 source meter and 532 nm continuous laser were used to study the light and dark photocurrent with variable sizes of the Cu mask on devices and laser intensity dependence. The photocurrent due to different incident wavelengths was analyzed by using grating with a xenon lamp source and a lock-in amplifier. Two probe conductivity measurements were performed using a Keithley Parameter Analyzer 4200SCS. The thickness of the HTL, active layer and ETL was measured using a 300 kHz tip in noncontact mode atomic force microscopy. The lifetime measurements were performed using a YSL photonics supercontinuum laser source. Scanning electron microscopy images were taken by using a Zeiss Gemini FESEM 500.

## 4 Results and discussion

### 4.1. Device structure

**Fig. 1:**
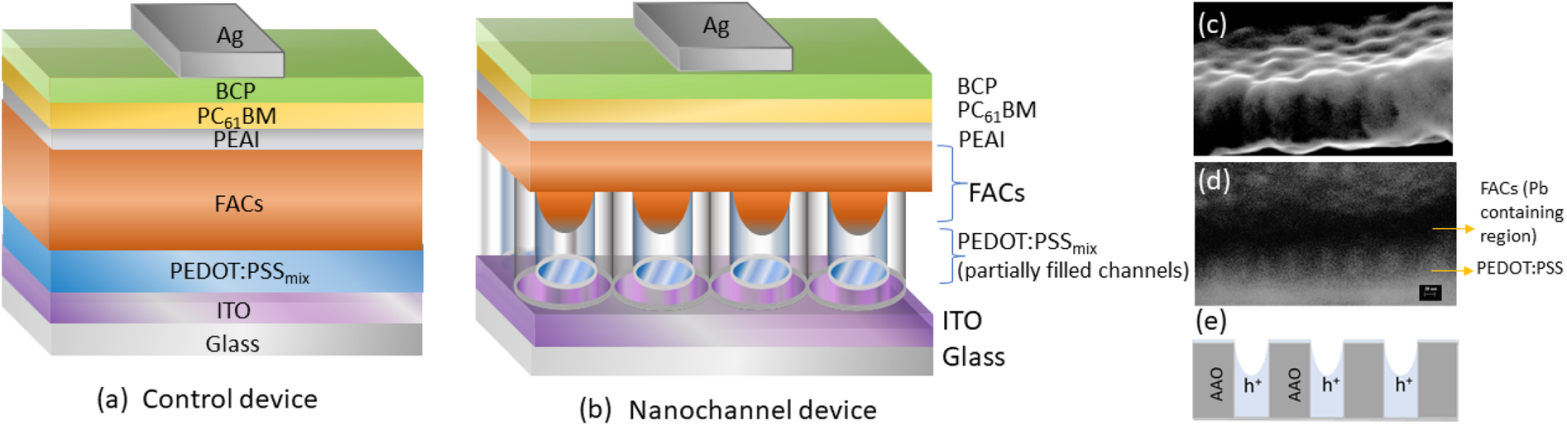
(a) Schematic structure of the control device and the (b) nanochannel HOIP device. The alumina nanochannels are partially filled with PEDOT:PSS and FACs (thickness of layers are not to scale). (c) Cross sectional SEM image of 20 nm AAO filled PEDOT:PSS and the FACs layers and (d) corresponding back scattered diffraction image revealing the filling of the lead containing (dark region) perovskite region through the length of alumina nanochannels. (e) shows a schematic of the hole transport path in nanochannel devices.

An inverted planar structure was incorporated as the HOIP bulk device shown in Fig. 1a. Noncontact mode atomic force microscopy (300 kHz tip) has been used to obtain the thickness of the spin-coated layers in photodetector devices. The approximate thicknesses of the thin films of PEDOT:PSS mix', FACs, PC_61_BM, BCP and electrode Ag layers are 60 ± 5 nm, 380 ± 35 nm, 80 ± 8 nm, 5 ± 2 nm and 120 ± 5 nm, respectively. HOIP nanochannel devices (Fig. 1b) are fabricated with an alumina template of 20 nm channel diameter. (ESI S6† shows the perovskite surface roughness). PEDOT:PSS mix' fills partial length ∼60 nm along the nanopillars. The perovskite layer fills the remaining length of the channels and an additional bulk layer is on top of these nanochannels. Nanoconfined PEDOT:PSS-filled nanochannels provide an excess surface area of occupancy for the perovskite crystal growth as compared to a similar bulk HOIP device area without alumina (the calculation of the approximate excess % area is shown in ESI S2†). Fig. 1c shows the cross-sectional SEM image of partially filled PEDOT/FACs in 20 nm AAO channels. The back-scattered diffraction image in Fig. 1e shows the lead-containing dark regions inside the channels and above the PEDOT layer.

Our previous reports^50,51^ have shown an increase in conductivity of PEDOT:PSS when confined in nanogeometries comparable to the transport length scales of the conducting polymer. The ordered domains and crystallinity of the nanoconfined polymers enhance the conductance of charge carriers along the nanochannel axes. This property of conducting polymer nanochannels is utilized to fabricate the nanochannel HOIP device. Usually the grade of PEDOT:PSS which contains a 1 : 6 ratio and has a work function of around 5.0 to 5.2 eV is generally used for photovoltaic applications. It has been earlier shown that the PH1000 grade of PEDOT:PSS which contains lower PSS content and work function around 4.8 to 5.0 eV is not suitable for a good amount of charge collection as it shows leakage current pathways. However, an optimized mixing of these two commercial grades can show improved behavior of the device performance. The two-probe IV characterization gives a high conductivity magnitude of 20 nm PEDOT:PSS mix' nanochannels with *σ*_transverse_ ∼3.6 (±1.1) × 10^−3^ S cm^−1^. The surface morphology, wettability and transmittability of these two PEDOT:PSS types are shown in ESI S3.† The optimal growth and grain size of the perovskite films are dependent on the hydrophilicity of the underlying films which is the PEDOT:PSS HTL in the present case. Nonwetting surfaces have larger grains, thus smaller trap densities. Since the hydrophilicity of the PEDOT:PSS mix' is slightly less than that of pristine PEDOT:PSS, the FAC grains in contact with the HTL might be larger than in the former device which might be responsible for one of the factors for improved responsivity. However, the top surface of FACs doesn't show much change in grain size. The crystallinity of the two variations of PEDOT:PSS nanochannels using high resolution transmission electron microscopy is also shown in ESI S4.†

### 4.2. Electrical characterization and spectral analysis

Upon illuminating the HOIP device from the ITO side under open-circuit conditions, a photovoltage is developed across the interface *V*_PV_ = *V*_light_ − *V*_dark_. The dark current essentially is the leakage current which originates from any trapped charge carriers or charge injection under reverse bias conditions. It is related to the crystal defects that result in the nonradiative recombination losses. The interface between the perovskite layer and the adjacent contact layers is important to analyze for reduced defects and match the energy levels. The electron transport layer PC_61_BM also plays an important role in passivating the charge defects and helps in reducing the current–voltage hysteresis due to trapped charges. Defects also get mitigated to some extent by adjusting the stoichiometric ratio of perovskite. Excess lead halide in the films has been shown to reduce nonradiative recombination significantly and thus improve the device performance.^53^ In this work, FA_0.95_Cs_0.05_Pb(I_0.83_Br_0.17_)_3_ stoichiometry has been found optimal for device performance. The HOIP photodetector device can be characterized by several figures of merit. A few of the parameters discussed below are photoresponsivity, external quantum efficiency, detectivity and response speed.

The efficiency of the device's response to the light signals is given by a critical parameter called photoresponsivity. It is the ratio of the effective photocurrent generated by the photodetector due to the photon absorption and the incident light power, *P*_in_ and is given by:i
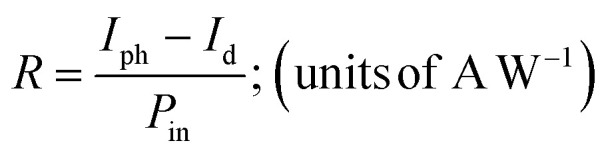
where *I*_ph_ is photocurrent under illumination and the *I*_d_ is the dark current. The high value of the responsivity means the device can convert optical signals to electrical ones efficiently.

External quantum efficiency (EQE) indicates the sensitivity of the device. A part of the incident photons is absorbed to generate free electrons and holes. Again, due to recombination losses and trap defects, only a part of these photogenerated carriers can generate photocurrent. This limit of the device is quantified by EQE:ii
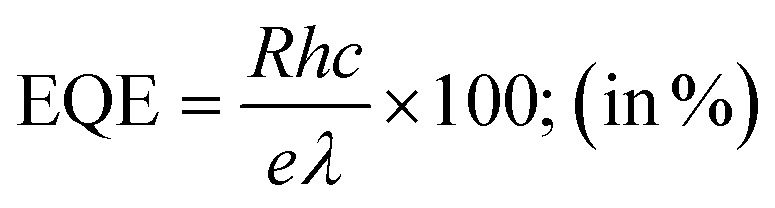
where *h* is Planck's constant, *e* is the electronic charge, *c* is the velocity of light in vacuum and *λ* is the incident light wavelength.

Specific detectivity which is another critical parameter can precisely reflect the limit of detecting light of lower intensities and is given by:iii
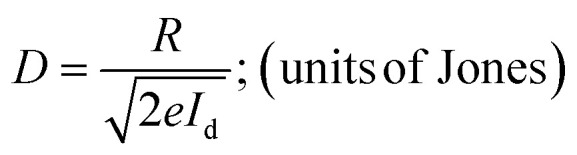


**Fig. 2:**
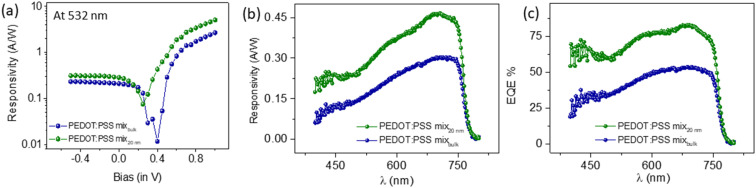
Electrical characterization. (a) Responsivity of the HOIP nanochannel and bulk device at 532 nm light source. (b) Responsivity and (c) EQE, respectively of the HOIP nanochannel and bulk devices over the spectral range.

The *I*–*V* characteristics of the HOIP device obtained through the incidence of a continuous laser (532 nm) are depicted in Fig. 2a. A circular mask is used at the ITO side of the device to maintain the same area for all devices (Experimental setup shown in ESI S7†). The responsivity of bulk and nanochannel devices turns out to be 0.21 A W^−1^ and 0.29 A W^−1^, respectively, and the corresponding dark currents of the devices are *I*_d_(bulk) = 50 nA and *I*_d_(20 nm) = 21 nA. EQE values are 48.9% and 67.5%, respectively, for bulk and nanochannel devices. Specific detectivity is of the order 1.6 × 10^12^ Jones and 3.5 × 10^12^ Jones, respectively, for bulk and nanochannel devices from eqn (iii). For the voltage range −0.5 V < *V* < 1.0 V, higher magnitudes of responsivity are observed for nanochannel devices. The dark currents of 20 nm channel devices are lower than those of the bulk devices. Dark currents in terms of series resistances and effective contributing electrode area are elaborated in ESI S2.†

The responsivity and EQE measured over a wide spectrum of 400 nm < *λ* < 800 nm have been shown in Fig. 2b and c. Xenon lamp is used as a broadband light source which is coupled to a monochromator to produce a near monochromatic beam of light and a lock-in amplifier records the photocurrent signals from the HOIP device connected through a 50 Ω coupler. A mask with a 2 mm diameter hole is used before the devices on the ITO side to keep the incident illumination area the same for all devices. The photocurrent signal from a Si photodetector is used for calibration and all the measurements have been performed in a closed dark chamber. Using eqn (i)–(iii), at *λ* = 532 nm, PEDOT:PSS mix' bulk devices have transport numbers *R* = 0.18 A W^−1^, EQE = 41.9% and *D* = 1.4 × 10^12^ Jones, while for the nanochannel devices, *R* = 0.28 A W^−1^, EQE = 65.2% and *D* = 3.4 × 10^12^ Jones. Fig. 2b and c show higher magnitudes of responsivity and EQE at all wavelengths for nanochannel devices of the PEDOT:PSS mix' as compared to the bulk devices. The transport numbers are similar to those obtained while using laser light of 532 nm (Fig. 2a). The possible effect of moisture in nanochannel devices has been discussed in ESI S5.†

**Fig. 3:**
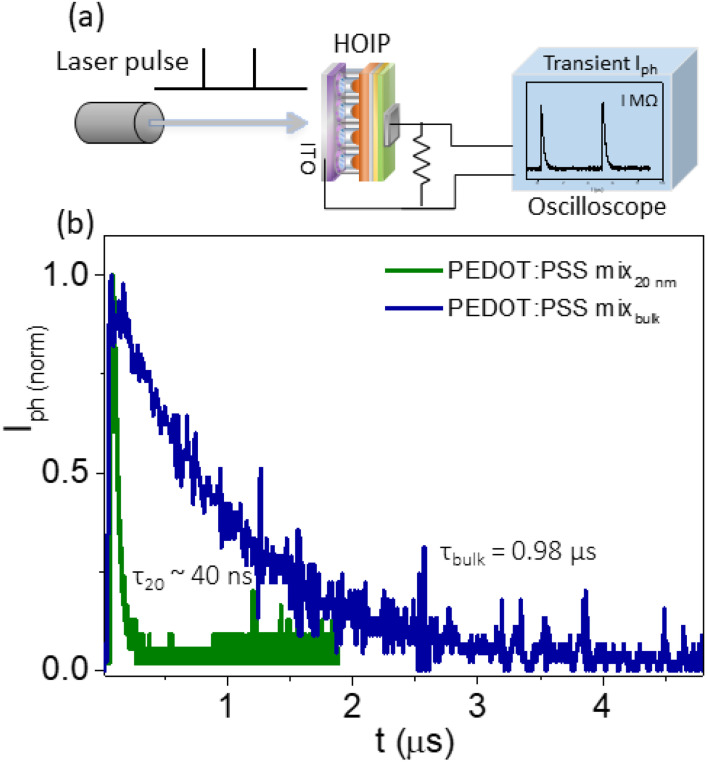
Transient photocurrent measurement. (a) Experimental setup using a pulsed supercontinuum laser source. (b) The response time, *τ* at the falling edge for HOIP bulk and nanochannel devices.

The response speed of the detectors is determined by the fall time, *τ* of the transient photocurrent to drop to 37% of its stable magnitude. Higher speed means shorter RC time constants and thus improved photodetector sensitivity. The response speed of the device is governed by the mobility of light-sensitive carriers and the effective distance between the two electrodes. Perovskites, being a direct bandgap material, have a high absorption coefficient and show high mobility of photogenerated carriers. Thus, enabling fast charge extraction leads to a fast response photodetector. Thus, a fast response photodetector is observed. In analyzing the small time taken for the carriers to travel the effective interelectrode distance, the smaller device area with lower capacitance showed the required RC time constant.^54^ A supercontinuum white light pulsed laser is used as a light source and triggered at the falling edge of the voltage pulse. The output signal is read from the oscilloscope. Fig. 3 shows shorter response time *τ* ∼ 40 ns for nanochannel HOIP while the bulk devices show *τ* ∼ 0.98 μs, which is nearly more than an order of magnitude higher than that of PEDOT nanochannel devices. The lower resistive PEDOT channels are mainly responsible for the faster time in nanochannel devices. The lower RC values for HOIP nanochannel devices give lower time scales and hence a faster response speed. Analyzing the transport parameters of the HOIP photodetector indicates that the nanochannel device performs more efficiently than their respective bulk devices.

## 5 Conclusion

A hybrid perovskite-based perovskite device incorporating a PEDOT:PSS-filled alumina scaffold has provided a platform to tune the electrical transport of charge carriers. The *I*(*V*) characteristics under dark and light conditions, EQE, detectivity and response timescales indicate improved performance parameters as compared to devices without the nanopores. This device architecture can open ways to achieve switching timescales in the sub-nanoseconds under reverse bias. The high conduction in confined PEDOT:PSS can be appropriately used in significantly improving performance in solar cells, batteries and light emitting diodes.

## Conflicts of interest

The authors declare no competing financial interest.

## Abbreviations

PEDOT:PSS: poly(3,4-ethylenedioxythiophene):poly(styrenesulfonate)
CPs: Conducting polymers
ITO: Indium tin oxide
AAO: Anodized aluminum oxide
HRTEM: High resolution transmission electron microscopy

## Supplementary Material

NA-005-D3NA00485F-s001
