# Supplementary material for: Structured hybrid photodetectors using confined conducting polymer nanochannels
Source: Nanoscale Adv. 2023 Oct 18;5(22):6155–61. doi: 10.1039/d3na00485f (PMC10628986; doi:10.1039/d3na00485f)
Supplement: NA-005-D3NA00485F-s001 [file NA-005-D3NA00485F-s001.pdf]

Electronic Supplementary Material (ESI) for Nanoscale Advances.
This journal is © The Royal Society of Chemistry 2023

Supplementary material

Structured hybrid photodetectors using confined conducting polymer nanochannels

*Sukanya Das, K.H. Girish, N. Ganesh, K. S. Narayan**

Chemistry and Physics of Materials Unit and School of Advanced Materials, Jawaharlal Nehru Centre for Advanced Scientific Research, Bengaluru- 560064, India.

*Email: narayan@jncasr.ac.in

1. Device architecture and SEM image of 20 nm AAO.
2. The calculation of effective surface area of nanochannel device in dark and light condition.
3. The surface morphology, optical transmission and contact angle measurements of PEDOT:PSS mix' and PEDOT:PSS
4. The crystallinity of the two types of PEDOT:PSS in nanochannels using high resolution transmission electron microscopy
5. Moisture effect on the nanochannel device
6. FACs roughness
7. Experimental setup used for measuring the photocurrent and voltage characteristics.

1. Schematic of device architecture and SEM image of 20 nm AAO.

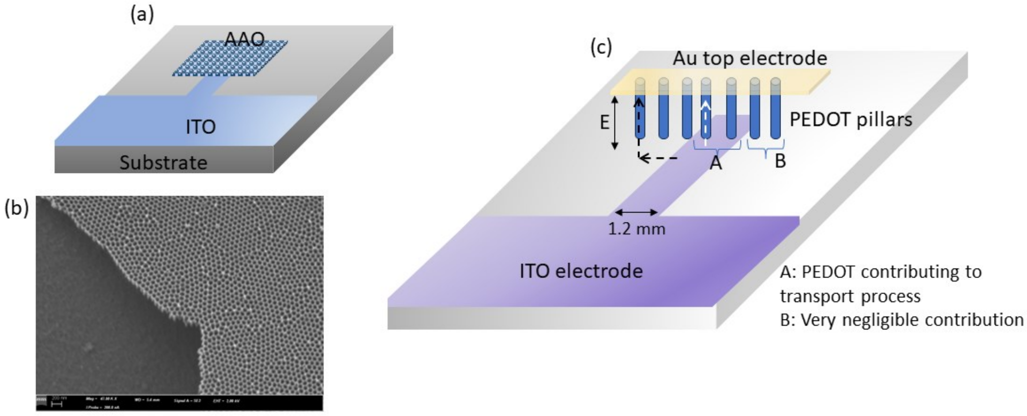

Figure S1. (a) Schematic of device structure. (b) SEM image of 20 nm AAO template and (c) schematic of ITO substrate to distinguish the contribution of PEDOT/AAO area in device characterization.

The ITO is patterned in the shape shown in (a) such that the AAO template is larger than the ITO width. Any excess PEDOT:PSS/FACs which retains during the spin coating on the broader ITO region away from AAO has been removed with cotton swab before measurements. The ITO substrates have been etched in a particular shape due to specific reasons described in Figure S1(c). The width of ITO at the centre region of interest is ~ 1.2 mm. The dimensions of AAO placed on ITO are usually 4*4 mm^2, which are quite larger than the ITO underneath.

Let the nanocylinders of alumina be named as 'A' type which are on the ITO and the nanocylinders on the glass (surrounding the ITO) be named as 'B' type. The larger size of AAO in comparison to ITO was to ensure: (i) proper adhesion of AAOs on the ITO and surrounding glass region and (ii) negligible seepage of the spin coated HTL layer through the periphery of AAO towards the ITO. This optimizes for the maximum transverse conduction and minimum

lateral contribution. The effective contributing current follows the white marked line and no-current from black line in S1(c). Only the PEDOT nanopillars of ‘A’ type contributes to current.

2. The calculation of effective surface area of device in dark and light condition

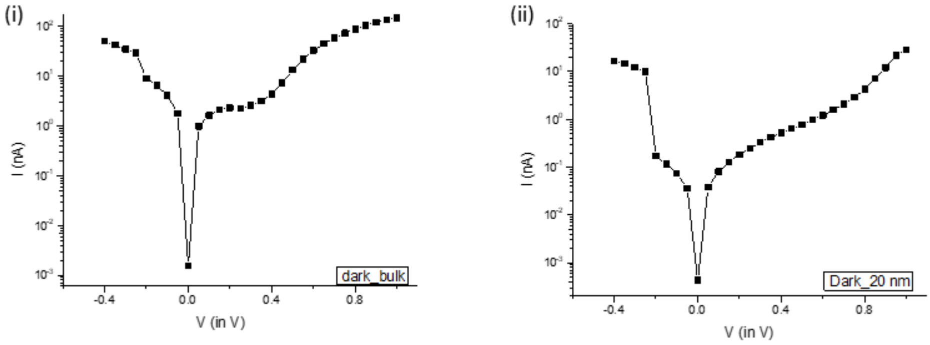

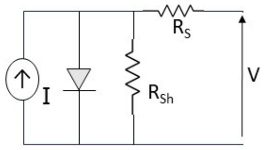

(a) Photodetector circuit

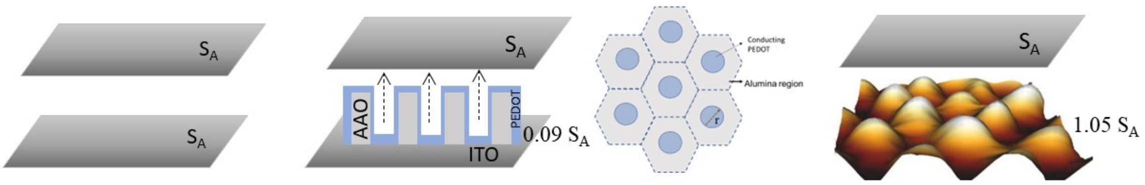

(b) Bulk device

(c) Nanochannel device (dark)

(d) Nanochannel device (light)

(e) The asymmetric interface (at Reverse bias)

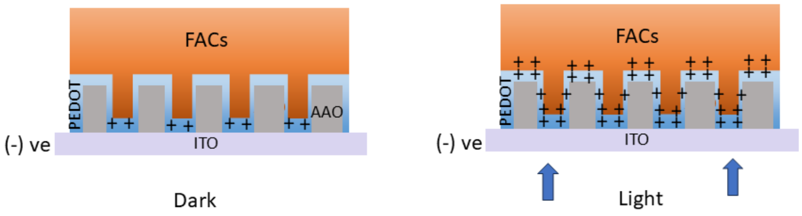

Figure S2. (i-ii) Dark IV features. (a) Schematic of photodetector circuit. (b,c,d) Effective surface area of the photodetector device at two electrodes in bulk, nanochannel device at dark and light conditions, respectively. The 3D surface morphology of PEDOT/FACs on AAO is represented as bottom surface in (d). (e) represents the transport at reverse bias conditions in 20 nm devices.

Series resistance R_s and shunt resistance R_{sh} plays important role in determining the device features. The device area of control device is assumed to be S_A. In dark conditions, $I_{d(20\ nm)}$= 18 nA and which is less than the bulk device current $I_{d(bulk)}$= 50 nA. This is because in absence of light the R_s in nanochannel devices is higher than the R_s of bulk device. Effective area of device in dark conditions is due to the effective HTL area in contact with ITO. For 20 nm devices, 91.41% of device area is solid alumina area. Thus, only 8.59% of area contributes for dark current. If the total surface area of control device, that is the planar bulk device is S_A, then effective area in dark conditions for 20 nm channel device is ~0.09 S_A. This is shown in Figure S2c. Under dark conditions, the extraction of holes in nanochannel devices are likely to take place only through PEDOT:PSS which is in contact with ITO due to the transverse transport (Figure S2(e)). (This is also prominent from the CAFM images provided from our earlier works[1]. The inter-channel conductivity is negligible. Prominent channels with high current are observed only from porous regions.) Thus, the effective area contributing to the resistance of whole device in dark conditions reduces to only 8.59% of the bulk device area.

In presence of light, the photoactive area is more in nanochannel device. The additional curved surface of the AAO template adds to the device area which contributes for transport. For 20 nm channels, the interpore diameter is 65 nm and interpore photoconductivity of charge carriers takes place over entire region of AAO/PEDOT/FACs. This is shown in Figure S2(d) and for 20 nm channels, taking into account the additional conical surface area of PEDOT/FACs the effective area is 5% more than the bulk area, i.e., effective area= 1.05 S_A. The photogenerated charge carriers are present all through curved surface area of PEDOT/FACs (Figure S2(e)). Due to the low resistances of PEDOT nanochannels and good interface of pedot/perovskite the effective area during light on conditions are higher than bulk surface area. Also, the extraction of charges takes place at a faster rate than in control devices, I=dQ/dt. This results in higher responsivity in nanochannel devices than in control devices.

3. The surface morphology, transmittability and contact angle measurements of PEDOT:PSS mix' and PEDOT:PSS.

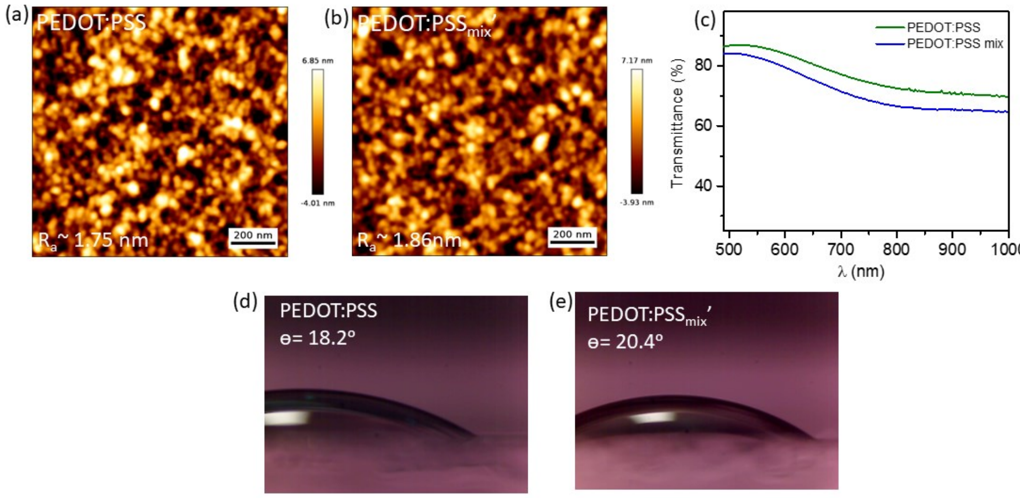

Figure S3: (a,b) Surface morphology of PEDOT:PSS and PEDOT:PSS mix' films and (c) represents the optical transmittability of two bulk thin films, respectively. (d,e) contact angle measurements of the respective PEDOT surfaces.

The two types of PEDOT films shows nearly similar surface roughness features with a slight higher average roughness (R_a ~ 1.86 nm) and lower wettability (higher ϴ= 20.4°) for PEDOT:PSS mix' bulk thin film surfaces.

4. The crystallinity of the two variations of PEDOT:PSS in nanochannels using high resolution transmission electron microscopy

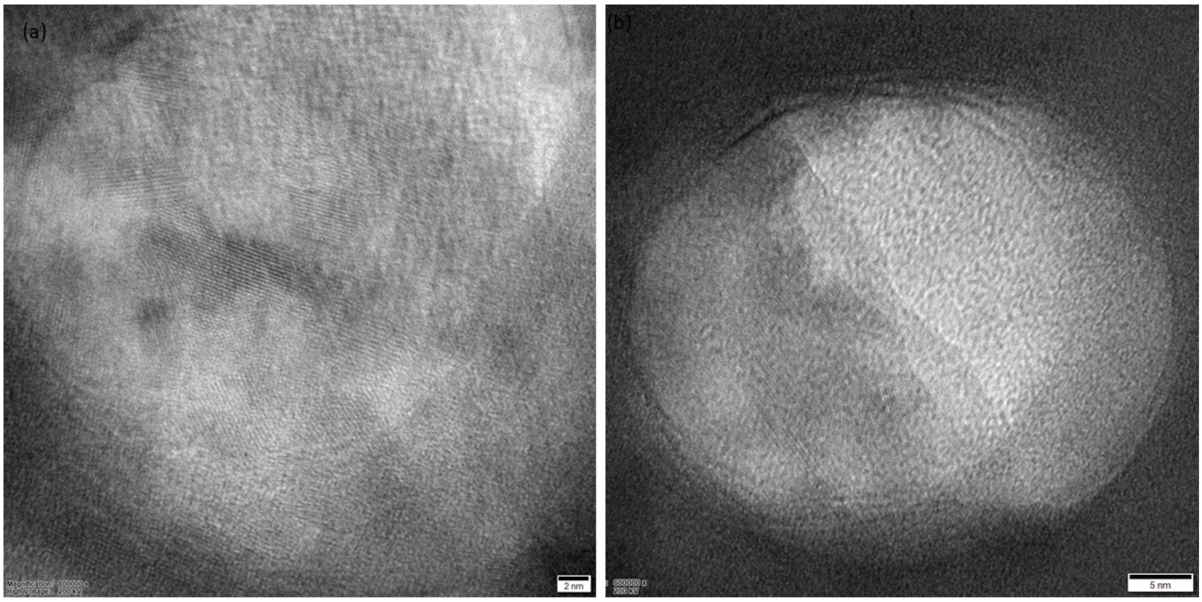

Figure S4: HRTEM images of PEDOT:PSS and PEDOT:PSS mix' films in 20 nm channels. Both the PEDOT films in nanochannels show interference fringes and polycrystallinity.

5. Moisture effect on the nanochannel device

The channel boundaries of alumina help in decreasing the ingression of water and moisture from the periphery towards the PEDOT/FACs. The stability of the nanochannel devices has been studied only for 28 days and retain their responsivity magnitudes. However, the bulk devices have shown to retain the responsivity magnitudes over approximately 19-20 days.

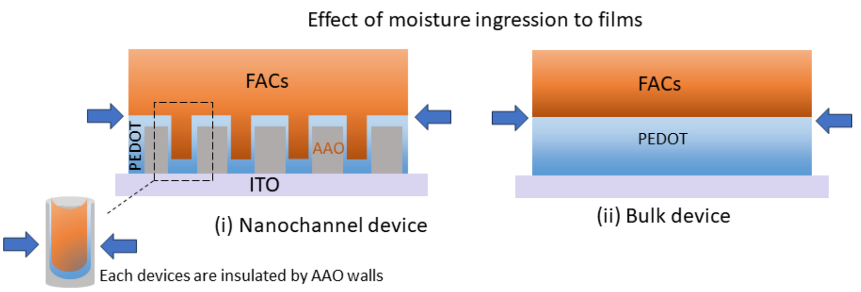

Figure S5. Schematic of device architecture and water or moisture ingression to films.

The transport parameters of the devices get affected with moisture and water due to absorption by PEDOT and perovskite layer. However, the architecture of alumina prevents moisture ingression through the periphery of films inwards. Each nanochannels containing PEDOT/FACs are well encapsulated by the boundary walls of alumina from the sides. This is in comparison to the bulk devices which are exposed to moisture from the lateral sides along the thickness of films. This is a possible reason for the stability of nanochannel devices over a longer duration.

6. FACs surface roughness

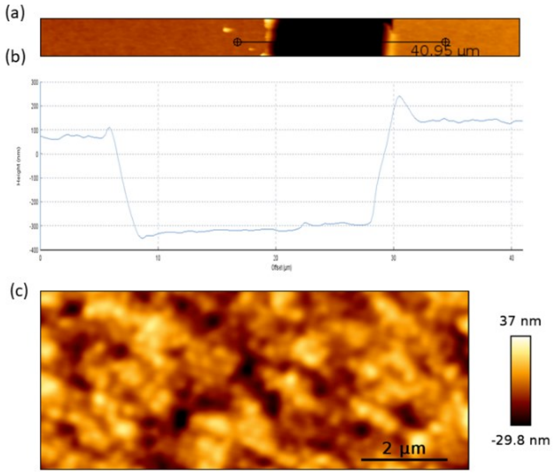

Figure S6. Surface of FACs on ITO/PEDOT. A cut made across FACs surface for thickness determination. (b) Line profile across FACs/PEDOT. (c) Top surface roughness of FACs.

Figure (a,b) shows thickness of composite film of PEDOT/FACs. The approximate thickness of FACs layer is ~ 380 nm.

7. Experimental setup used for measuring the photocurrent and voltage characteristics

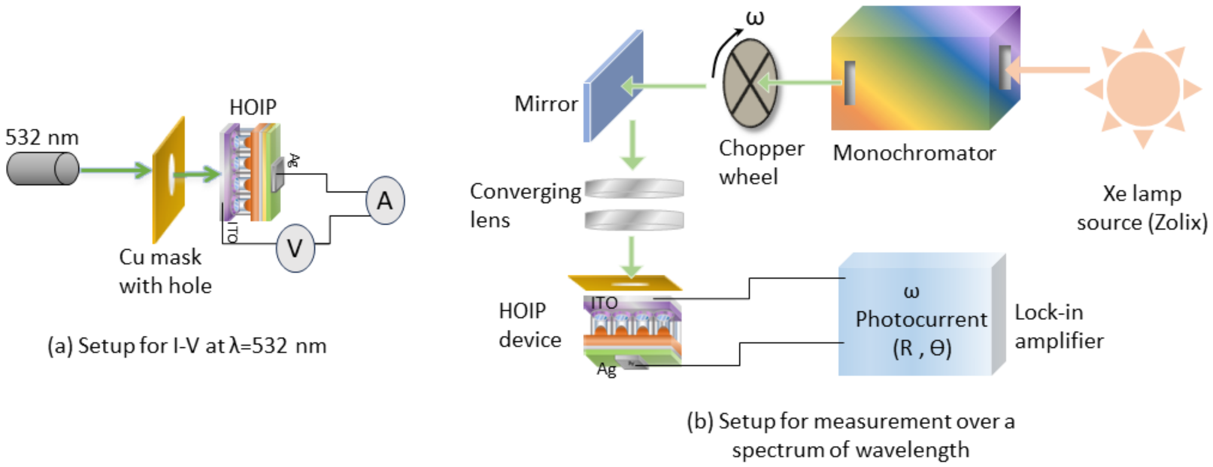

Figure S7. Experimental setup for I-V characterization at (a) 532 nm and (b) over range of wavelength.

The experimental setup used for photocurrent and voltage characteristics are shown in Figure S7. For the responsivity measurements, a copper mask with a small hole (diameter~2 mm) has been used before the sample on ITO side to avoid collection of photocurrent from adjoining areas of PEDOT/FACs as shown in Figure S7(a). The size of the hole exposes a region which contains only AAO/PEDOT/FACs and not the region without alumina. The power of the laser is measured using a power meter (with silicon detector) using the same circular mask held infront of the detector for normalization with laser power in calculation of responsivity. Same mask is used for the measurement with Xe lamp light through a monochromator for spectral analysis. The mask is held fixed against ITO side of the sample during the measurements at all wavelengths (Figure S7(b)). EQE= $Rhc/e\lambda$ *100% has been obtained in Figure 2c in manuscript by normalizing the respective responsivity (and the factor hc/e) with the corresponding wavelength values.

References

1. Das, S. and K.S. Narayan, *Significant Increase in Electrical Transport of Conducting Polymers Confined in Alumina Nanopores.* The Journal of Physical Chemistry C, 2019. **123**(17): p. 11284-11291.
